# Ferroptosis in Glioblastoma and Neuroblastoma: Molecular Mechanisms and Novel Therapeutic Strategies

**DOI:** 10.3390/cimb48030267

**Published:** 2026-03-03

**Authors:** Zhaoyang Liu, Zihan Ma, Kexin Yang, Hongwei Fan

**Affiliations:** 1 Department of Clinical Pharmacology Lab, Nanjing First Hospital, Nanjing Medical University, Nanjing 210006, China; lzy072411@163.com (Z.L.); mazihan82024@163.com (Z.M.); 2 School of Basic Medicine and Clinical Pharmacy, China Pharmaceutical University, Nanjing 211122, China; yangkx0430@163.com

**Keywords:** ferroptosis, glioblastoma, neuroblastoma, tumor immune microenvironment, targeted therapeutic strategies

## Abstract

Malignant neoplasms arising from the central nervous system (CNS), particularly glioblastoma (GBM) as well as neuroblastoma (NB), represent a formidable global health burden owing to their aggressive biological behavior and dismal clinical outcomes. Ferroptosis—an iron-dependent form of regulated cell death distinct from apoptosis, autophagy, and necrosis—has emerged as a critical regulatory nexus in the progression and therapeutic response of these malignancies. Characterized by iron-catalyzed lipid peroxidation, ferroptosis is tightly governed by the metabolic interplay among lipids, iron, and glutathione, profoundly influencing tumorigenesis, tumor progression, and therapeutic resistance. In this review, we systematically synthesize current knowledge on ferroptosis in GBM and NB, specifically contrasting how developmental origins and metabolic contexts shape their regulatory mechanisms. We further integrate recent advances in the diagnostic and therapeutic landscape of nervous system tumors, with a particular emphasis on ferroptosis-targeted strategies. Overall, this work aims to provide a conceptual framework linking ferroptosis regulation to tumor context, thereby offering mechanistic insights and future directions for the precision management of nervous system malignancies.

## 1. Introduction

Malignant neoplasms of the central nervous system (CNS) remain among the most challenging cancers worldwide. Surveillance data indicate that approximately 85,000 individuals in the United States are diagnosed with primary brain tumors annually, with nearly 29% classified as malignant [1]. Despite decades of intensive research and therapeutic advances [2,3], epidemiological projections predict a continued rise in mortality from brain and other nervous system cancers by 2040 [4], underscoring a substantial and growing public health burden.

Glioblastoma (GBM), the most prevalent malignant primary brain tumor in adults, exhibits increasing incidence after 40 years of age and peaks in the elderly population [5]. GBM is distinguished by its highly aggressive biological behavior, profound inter- and intratumoral heterogeneity, nearly universal recurrence, and extensive metabolic reprogramming that supports therapeutic resistance [6]. Despite the standard Stupp protocol, incorporating maximal surgical resection followed by concomitant radiochemotherapy, median overall survival remains limited to 12–15 months [7].

Neuroblastoma (NB) arises from neural crest-derived progenitor cells, and represents the most common extracranial solid tumor in children [8]. Although low-risk NB demonstrates favorable outcomes, high-risk variants remain refractory to multimodal therapy, account for a disproportionate fraction of childhood cancer-related mortality, and exhibit high rates of disease recurrence, reflecting marked clinical heterogeneity with distinct metabolic and redox states relevant to ferroptosis susceptibility [9,10]. MYCN (MYCN proto-oncogene, bHLH transcription factor) amplification and profound metabolic plasticity are hallmark features of high-risk NB and contribute to treatment resistance and disease relapse.

Regulated cell death (RCD) pathways are essential for tissue homeostasis. Among them, ferroptosis has gained considerable attention as a distinct non-apoptotic cell death modality driven by iron-dependent lipid peroxidation [11]. Ferroptosis occurs when dysregulated iron metabolism or imbalance between lipid oxidative damage and thiol-dependent antioxidant systems leads to excessive accumulation of reactive oxygen species (ROS) [12]. Ferroptosis functions as an important tumor-suppressive mechanism and has emerged as a therapeutic target across multiple malignancies, including non-small cell lung cancer, hepatocellular carcinoma, pancreatic ductal adenocarcinoma, and triple-negative breast cancer [13,14,15,16]. Growing evidence implicates ferroptosis in the progression and treatment response of nervous system tumors, highlighting it as a potential therapeutic vulnerability [17,18].

Several recent studies have summarized ferroptosis mechanisms and therapeutic methods in cancer, including nervous system tumors, providing systematic overviews of ferroptosis signaling networks and metabolic regulation [19,20]. However, how ferroptosis regulation is systematically shaped by tumor-specific developmental origin, metabolic wiring, and microenvironmental constraints remains incompletely resolved. In this context, GBM and NB represent two clinically aggressive yet biologically distinct nervous system malignancies, differing fundamentally in cellular lineage, age of onset, oncogenic drivers, metabolic dependencies, and immune landscapes. Here, we focus on GBM and NB as representative adult and pediatric nervous system tumors to highlight how ferroptosis regulation diverges across developmental contexts. Where appropriate, we contrast these tumor-specific features with ferroptosis regulation in other malignancies and in normal neural tissues, in order to emphasize disease-specific vulnerabilities and therapeutic implications. By synthesizing recent mechanistic and translational advances across both tumor types, this review aims to elucidate the roles of ferroptosis in diagnosis, therapeutic resistance, and emerging treatment strategies, and to outline a conceptual framework to inform future ferroptosis-based research in nervous system malignancies.

## 2. Biological and Cellular Features of GBM and NB Relevant to Ferroptosis

### 2.1. Molecular and Metabolic Characteristics of GBM Predisposing to Ferroptosis Regulation

GBM exhibits pronounced cellular plasticity and metabolic reprogramming, creating a context in which ferroptosis is closely linked to tumor survival and therapy resistance [21]. This state is defined by dysregulated iron homeostasis, altered lipid metabolism, and a strong dependence on antioxidant defense systems, which together converge on ferroptosis regulation [22].

Lipid metabolic reprogramming sensitizes GBM cells to ferroptosis by increasing the incorporation of polyunsaturated fatty acids (PUFAs) into membrane phospholipids via enzymes such as ACSL4 (acyl-CoA synthetase long-chain family member 4) and LPCAT3 (lysophosphatidylcholine acyltransferase 3) [23]. PUFA-enriched membranes constitute preferred substrates for lipid peroxidation, rendering ferroptosis a latent vulnerability. However, this vulnerability is counterbalanced by a pronounced reliance on GPX4 (glutathione peroxidase 4)-mediated lipid peroxide detoxification, reinforcing GPX4-dependent ferroptosis resistance in GBM [19].

Iron metabolism is extensively remodeled in GBM. Upregulation of transferrin receptor 1 (TFR1), increased ferritin turnover, and enhanced ferritinophagy collectively expand the labile iron pool (LIP), thereby amplifying iron-dependent oxidative stress [20]. Rather than passively undergoing ferroptosis, GBM cells exhibit active mechanisms to restrain iron toxicity through reinforced antioxidant pathways, with GPX4 functioning as a key downstream effector that integrates iron-driven lipid peroxidation signals with cell survival.

The GBM tumor microenvironment (TME) further modulates ferroptosis sensitivity. Hypoxia, metabolic competition, and oxidative stress signaling shape redox balance and iron availability, while pathways such as NRF2 (nuclear factor erythroid 2 related factor 2) and STAT3 (signal transducer and activator of transcription 3) converge on the transcriptional and metabolic control of GPX4 and related antioxidant defenses. Collectively, these features suggest that ferroptosis in GBM is tightly constrained by GPX4-dominated resistance mechanisms [24].

### 2.2. Developmental Origin and Ferroptosis-Related Vulnerabilities in NB

NB exhibits a distinct biological architecture shaped by developmental programs rather than accumulated somatic mutations. This developmental origin profoundly influences ferroptosis regulation, rendering NB uniquely dependent on mitochondrial metabolism, redox balance, and iron handling.

High-risk NB is characterized by MYCN oncogene amplification, which drives widespread metabolic rewiring. MYCN-driven transcriptional programs enhance oxidative phosphorylation and nucleotide and lipid biosynthesis, thereby increasing intracellular ROS generation. While this metabolic state supports rapid proliferation, it simultaneously creates intrinsic vulnerability to oxidative damage and ferroptosis if glutathione-dependent redox buffering is compromised, positioning GSH (glutathione) availability—rather than GPX4 abundance per se—as a critical determinant of ferroptotic sensitivity [25,26].

Iron metabolism in NB is closely coupled to developmental and mitochondrial demands. Elevated iron uptake and altered expression of iron-handling proteins such as transferrin receptor and ferroportin modulate intracellular iron availability, influencing mitochondrial function and lipid peroxidation dynamics. Unlike GBM, where ferroptosis resistance is often reinforced through GPX4-centric mechanisms, NB cells—particularly MYCN-amplified subtypes—may exhibit heightened ferroptosis sensitivity due to their strong reliance on GSH-mediated redox homeostasis to restrain oxidative stress [27].

NB also displays distinctive amino acid and lipid metabolic dependencies, including altered glutamine utilization and cholesterol biosynthesis, which critically support intracellular GSH synthesis and recycling [28].

### 2.3. Rationale for Focusing on GBM and NB as Complementary Ferroptosis Models

Taken together, GBM and NB represent two biologically distinct yet clinically aggressive nervous system malignancies that exemplify the context-dependent nature of ferroptosis regulation. GBM reflects an adult tumor paradigm marked by extreme heterogeneity, adaptive ferroptosis resistance, and microenvironment-driven metabolic flexibility. In contrast, NB represents a pediatric, developmentally rooted malignancy in which ferroptosis sensitivity is tightly coupled to mitochondrial metabolism and oncogene-driven redox stress.

By examining these tumors in parallel, it becomes possible to delineate both shared ferroptosis regulatory cores and tumor-specific modulatory layers shaped by developmental origin, metabolic wiring, and immune context. Notably, this comparison highlights GPX4-centered ferroptosis resistance in GBM versus GSH-centered redox dependency in NB, providing a conceptual framework for tumor- and context-specific ferroptosis targeting [29]. Importantly, compared with other solid tumors and normal neural tissues, GBM and NB display distinct ferroptosis regulatory architectures shaped by extreme metabolic stress, developmental programs, and lineage-specific redox constraints, underscoring their value as complementary disease models [30].

## 3. Core Ferroptosis Mechanisms and Their Tumor-Context Dependency

**Figure 1:**
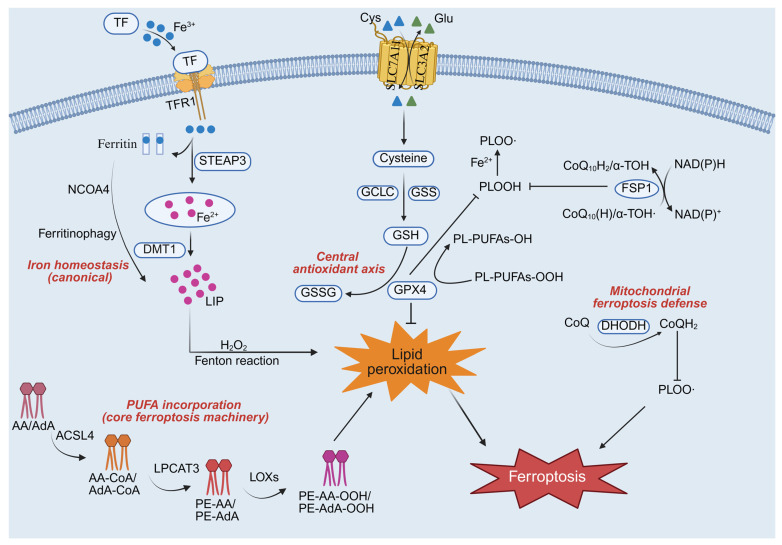
Canonical ferroptosis machinery shared across cell types. ACSL4 and LPCAT3 mediate the incorporation of PUFAs into PL-PUFAs. These PL-PUFAs are then peroxidized to drive lipid peroxidation and ferroptosis. Intracellular Fe^2+^, sourced from transferrin-bound Fe^3+^ or ferritinophagy, constitutes the LIP. Overload of the LIP promotes the Fenton reaction, which drives lipid peroxidation and triggers ferroptosis. Cystine uptake via the transporter SLC7A11 provides cysteine for GSH synthesis. GSH then acts as a cofactor for GPX4 to reduce PL-PUFA-OOH to PL-PUFA-OH, thereby inhibiting ferroptosis. In mitochondria, CoQ is reduced to CoQH_2_ under the action of DHODH, and this process inhibits the generation of PLOO radicals. Abbreviations: TF, transferrin; TFR1, transferrin receptor 1; STEAP3, iron oxide reductase steam 3; DMT1, divalent metal transporter 1; NCOA4, nuclear receptor coactivator 4; LIP, labile iron pool; AA, arachidonic acid; AdA, Adrenic acid; ACSL4, acyl-CoA synthetase long-chain family member 4; LPCAT3, lysophosphatidylcholine acyltransferase 3; PE, phosphatidylethanolamine; LOXs, lipoxygenases; Cys, cystine; Glu, glutamate; SLC7A11, solute carrier family 7 member 11; SLC3A2, solute carrier family 3 member 2; GCLC, glutamate-cysteine ligase catalytic; GSS, glutathione synthetase; GSH, glutathione; GSSG, oxidized glutathione; GPX4, glutathione peroxidase 4; PLOOH, phospholipid hydroperoxide; PUFAs, polyunsaturated fatty acids; PL-PUFAs, phospholipid-containing PUFAs; α-TOH, α-Tocopherol; NADPH, nicotinamide adenine dinucleotide phosphate; DHODH, dihydroorotate dehydrogenase.

Although ferroptosis is governed by conserved biochemical pathways, its execution and therapeutic relevance are highly context-dependent in nervous system tumors. Here, we summarize key ferroptosis mechanisms (Figure 1) with a specific focus on how developmental origin, metabolic wiring, and redox control differentially shape ferroptotic vulnerability in GBM and NB.

### 3.1. Lipid Metabolism and Membrane Vulnerability in GBM and NB

Lethal lipid peroxidation of PUFA-containing phospholipids constitutes a central biochemical hallmark of ferroptosis. Iron-catalyzed oxidation of PUFA-enriched membrane lipids generates lipid hydroperoxides that compromise membrane integrity and trigger ferroptotic cell death [31,32].

Both nonenzymatic ROS-driven reactions and enzymatic lipid oxidation processes contribute to lipid peroxide accumulation [33,34]. In GBM, hypoxia-associated PUFA remodeling promotes lipid peroxidation but is offset by strong antioxidant defenses, whereas in NB, MYCN-driven metabolic programs differentially shape lipid composition and redox balance. These contrasts highlight membrane lipid composition as a tumor-context-dependent regulator of ferroptosis sensitivity.

### 3.2. Iron Metabolism: Distinct Iron Dependencies in Adult Versus Pediatric Tumors

Iron is indispensable for cellular metabolism but also serves as a potent catalyst of lipid peroxidation through Fenton chemistry. Dysregulated iron uptake, storage, and mobilization expand the cytosolic LIP, thereby amplifying oxidative stress and sensitizing cells to ferroptosis [35,36,37].

In GBM, dysregulated iron uptake and ferritinophagy expand the LIP, amplifying iron-driven lipid peroxidation under hypoxic stress. In NB, iron dependency shaped by developmental programs and MYCN-driven metabolism may lower the threshold for iron-dependent oxidative damage. These contrasts suggest that iron-handling pathways confer tumor-specific ferroptosis vulnerabilities rather than serving as uniform regulatory nodes across nervous system malignancies.

### 3.3. Antioxidant Systems and GPX4 Dependency in Nervous System Tumors

The GSH/GPX4 axis functions as a critical checkpoint restraining lipid peroxidation during ferroptosis. Disruption of this axis—via cystine deprivation, GSH depletion, or GPX4 inhibition—leads to unchecked lipid peroxide accumulation and ferroptotic death [38,39].

In GBM, persistent oxidative stress and PUFA-enriched membranes confer a strong dependence on GPX4 activity to suppress ferroptosis [29]. In contrast, NB cells rely primarily on sustained glutathione availability to maintain redox balance during rapid proliferation, rendering GSH metabolism a central determinant of ferroptotic sensitivity. These differences highlight context-dependent antioxidant dependencies in adult versus pediatric nervous system tumors.

### 3.4. GPX4-Independent Pathways and Emerging Tumor-Specific Regulators

Coenzyme Q (CoQ) plays a pivotal protective role against ferroptosis, primarily owing to its potent antioxidant properties. As a lipid peroxide radical scavenger, CoQ is crucial for preventing lipid peroxidation and the subsequent cell death that ensues. The ferroptosis suppressor protein 1 (FSP1) protein enhances CoQ’s antioxidant capacity by facilitating its NADH-dependent reduction, thereby inhibiting the initiation of ferroptosis [40].

Cells with impaired CoQ synthesis, such as those with FSP1 deficiency, exhibit increased susceptibility to ferroptosis. This underscores the significance of CoQ regeneration and its antioxidant functions in maintaining the integrity of cellular membranes [41]. Although FSP1 can confer protection within lipid droplets, its localization at the plasma membrane is of critical importance for its ability to counteract ferroptosis. Although the FSP1–CoQ axis has been primarily characterized in non-neural tumor models, emerging evidence suggests that this GPX4-independent defense may be particularly relevant in nervous system tumors with intrinsic resistance to GPX4 inhibition, warranting further investigation in GBM and NB contexts.

This mechanism underscores the therapeutic potential of targeting the FSP1-CoQ10 axis, particularly in tumors that have developed resistance to GPX4-targeted therapies, offering a complementary strategy to maximize ferroptotic cell death.

## 4. The Molecular Mechanisms of Ferroptosis in GBM and NB

Ferroptosis is not merely involved in the onset of neurological malignancies but also drives their progression via distinct regulatory pathways (Table 1).

### 4.1. Molecular Mechanism of Ferroptosis in GBM

**Figure 2:**
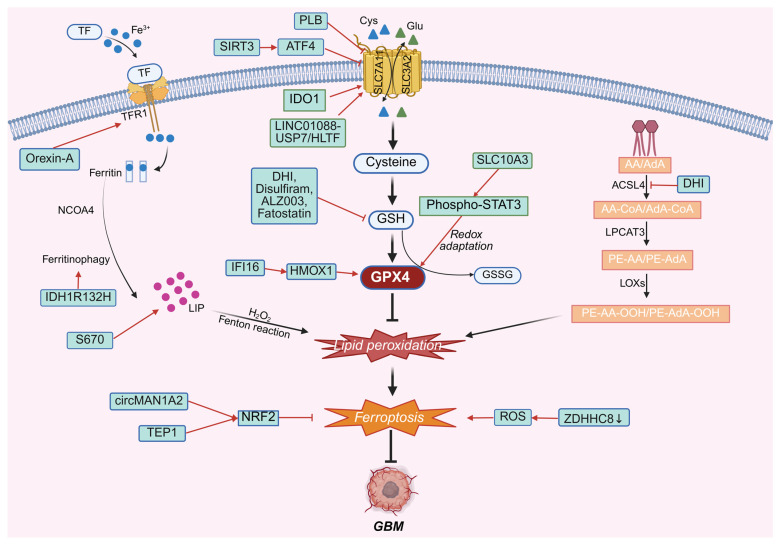
GBM-specific regulatory rewiring of ferroptosis. The regulation of ferroptosis can exert significant impacts on GBM through multiple mechanisms, encompassing modulation of glutathione levels, lipid metabolism, iron metabolism, as well as the NRF2 and STAT3 signaling pathways, with GPX4 functioning as a central integrative node linking these regulatory axes. Abbreviations: PLB, plumbagin; IDO1, indoleamine2,3-dioxygenase1; SIRT3, sirtuin3; ATF4, activating transcription factor 4; SLC10A3, solute carrier family 10 member 3; IFI16, interferon-inducible protein 16; HMOX1, heme oxygenase 1; ROS, reactive oxygen species; NRF2, erythroid-derived 2; TEP1, telomerase associated protein 1; DHI, 15,16-dihydrotanshinone I; HLTF, helicase-like transcription factor.

Within the uniquely heterogeneous and therapy-refractory landscape of GBM, ferroptosis is modulated through multiple mechanistic dimensions, including GSH metabolism, lipid oxidation, iron homeostasis, GPX4 activity, and dysregulation of antioxidant pathways such as NRF2 and STAT3 (Figure 2).

#### 4.1.1. GPX4/GSH Regulatory Axis

In GBM, GSH-dependent regulation of the system xc^−^/GPX4 axis critically shapes ferroptotic sensitivity.

Interferon-inducible protein 16 (IFI16) suppresses radiotherapy-induced ferroptosis in GBM by activating the HMOX1 (heme oxygenase 1)–GPX4 axis, thereby limiting lipid peroxidation and Fe^2+^ accumulation and promoting radiation resistance. Clinically, elevated IFI16 expression correlates with tumor recurrence and unfavorable prognosis, underscoring its role in ferroptosis-mediated therapeutic resistance [42]. Pharmacologically, disulfiram has been shown to induce ferroptosis and lysosomal membrane permeabilization in a ROS-dependent manner, sensitizing GBM cells to radiotherapy by blocking the xCT/GPX4 axis [43].

SLC7A11 (solute carrier family 7 member 11), a core component of system xc^−^, enhances cystine uptake, promotes GSH biosynthesis, and increases GPX4 enzymatic activity, collectively reducing ferroptotic vulnerability [67]. The zinc finger DHHC-type containing 8 (ZDHHC8) gene encodes a crucial and highly specific palmitoyltransferase that plays a pivotal role in the pathogenesis and progression of GBM. ZDHHC8 inactivation impairs SLC7A11 palmitoylation, precipitating lysosomal degradation of SLC7A11 [47].

In temozolomide-resistant GBM, the curcumin analog ALZ003 induces FBXL2 (F-box and leucine rich repeat protein 2)-mediated ubiquitination and degradation of the androgen receptor (AR), leading to GPX4 downregulation, elevated lipid peroxidation, and ferroptotic cell death [44]. Similarly, SIRT3 (sirtuin3) inhibition increases GBM susceptibility to RSL3-induced ferroptosis by downregulating SLC7A11 through ATF4 (activating transcription factor 4) activation, promoting mitophagy, and driving mitochondrial Fe^2+^ and ROS accumulation [45].

Fatostatin triggers ferroptosis by inhibiting the AKT/mTORC1 pathway, thus impairing GPX4 protein synthesis, elevating lipid ROS, and depleting intracellular GSH [46]. Emerging evidence suggests that non-coding RNAs constitute an additional regulatory layer modulating ferroptosis-related genes involved in redox homeostasis, although their roles in nervous system tumors remain incompletely defined [68]. Zhou et al. illustrated that long intergenic non-protein coding RNA 01088 (LINC01088) interacts with ubiquitin-specific protease 7 (USP7) and helicase-like transcription factor (HLTF), stabilizing HLTF to upregulate SLC7A11 [53]. This regulatory cascade potentiates GSH biosynthesis, sustains GPX4 enzymatic activity, and collectively confers ferroptosis resistance in GBM cells.

#### 4.1.2. Iron Metabolism

Elevated intracellular LIP levels represent a central driver of ferroptosis in GBM. Iron homeostasis integrates import, storage, trafficking, and autophagic degradation pathways that collectively regulate ferroptotic susceptibility.

Ferritinophagy, a well-characterized autophagic process, involves nuclear receptor coactivator 4 (NCOA4)-driven lysosomal degradation of ferritin, with the consequent liberation of sequestered iron. Shenoy et al. demonstrated that targeted inhibition of NCOA4 mitigates ferroptosis, presumably by diminishing the intracellular LIP [69]. Emerging evidence has unraveled that the IDH1R132H mutation triggers ferritinophagy-mediated iron metabolic reprogramming by suppressing the PRMT1-PTX3 epigenetic axis. This is marked by downregulated expression of ferritin (FTH1/FTL) and the iron exporter solute carrier family 40 member 1 (SLC40A1), alongside upregulated expression of the autophagic receptor NCOA4. These molecular alterations collectively induce free iron accumulation, ROS surge, and enhanced susceptibility to ferroptosis [70]. Pharmacological modulation also impacts iron homeostasis. For instance, the compound S670 precipitates iron overload and lipid peroxidation by diminishing ferritin heavy chain (FTH) expression [55], while Orexin-A has been found to upregulate transferrin receptor (TFRC) and downregulate FTH1, similarly expanding the LIP to trigger ferroptosis [57].

#### 4.1.3. Lipid Metabolism

Lipid remodeling critically governs ferroptosis execution by influencing ROS-mediated membrane destabilization.

Meng et al. [48] demonstrated that dysregulated lipid metabolism modulates ferroptosis in GBM via the C5aR1–ERK1/2–METTL3 signaling cascade. Activation of this pathway enhances N^6^-methyladenosine (m^6^A) modification and stabilizes GPX4 mRNA, thereby suppressing ferroptosis. Pharmacological inhibition of C5aR1 (C5a receptor) reverses this effect and sensitizes GBM cells to ferroptotic death . Zeng et al. [49] elucidated that tumor necrosis receptor-associated factor 3 (TRAF3) orchestrates the oxidation of PUFAs and lipid peroxidation via K63-linked ubiquitination of enoyl-CoA hydratase 1 (ECH1). Consequently, TRAF3 exerts regulatory influences on ferroptosis and metabolic reprogramming in GBM cells . Tian et al. [51] provided compelling evidence indicating that overexpression of indoleamine 2,3-dioxygenase 1 (IDO1) mitigates ferroptosis in GBM by augmenting the stability of SLC7A11 mRNA through m^6^A methylation. This regulatory pathway modulates lipid metabolic homeostasis and facilitates GBM progression.

Plumbagin (PLB), a natural quinone compound, downregulates SLC7A11/xCT and GPX4, reduces GSH levels, enhances lipid peroxidation, and induces ferroptosis, thereby suppressing GBM proliferation [52].

#### 4.1.4. Mitochondrial Metabolism

Mitochondria are integrally involved in the modulation of ferroptosis through their roles in redox homeostasis and iron handling. Dysregulation of mitochondrial function enhances oxidative stress and accelerates ferroptotic execution.

Recent investigations into ferroptosis regulation revealed that heat shock protein 27 (HSP27) depletion significantly alters cellular iron metabolism. Specifically, this manipulation facilitates ferrous iron uptake while suppressing the small ubiquitin-like modifier (SUMO)ylation of ACSL4, thereby enhancing its stability. Additionally, the process of lipid peroxidation is intensified, as evidenced by the elevated levels of malondialdehyde (MDA). These mitochondrial alterations collectively trigger ferroptotic pathways, ultimately leading to a deceleration in glioblastoma cell proliferation [56].

#### 4.1.5. NRF2 Signaling Pathway

Functional characterization revealed that circMAN1A2 depletion in GBM cells strengthens the TEP1-KEAP1 interaction, leading to enhanced KEAP1-dependent NRF2 ubiquitination and proteasomal degradation. Conversely, circMAN1A2 overexpression competitively binds TEP1, disrupting its complex with KEAP1 and stabilizing NRF2 [71]. This reciprocal regulation establishes a circMAN1A2/TEP1/KEAP1 axis that governs redox homeostasis in glioma genesis. Ferroptosis is characterized by elevated levels of ROS and MDA, a decreased content of GSH, and mitochondrial dysfunction. Moreover, this process can reverse TMZ resistance in glioblastoma stem cells (GSCs) [50].

Importantly, given the extreme oxidative stress and metabolic heterogeneity characteristic of GBM, NRF2-dependent antioxidant reinforcement constitutes a dominant barrier to ferroptosis execution, distinguishing GBM from tumors with weaker redox buffering capacity. Procyanidin B1-mediated inhibition of NRF2 elicits ferroptosis in GBM. Gao et al. [54] demonstrated that procyanidin B1 directly interacts with NRF2, promoting PSMC3-mediated ubiquitin-dependent degradation of NRF2 and impairing the cell’s capacity to scavenge H_2_O_2_. This sequence of events activates ferroptosis-related signaling pathways, which are characterized by elevated levels of Fe^2+^ and lipid hydroperoxide (LOOH), upregulated expression of cyclooxygenase-2 (COX2), and reduced expression of SLC7A11 in GBM cells.

#### 4.1.6. STAT3 Signaling Pathway

STAT3, a central component within the STAT family of transcription factors, acts as a crucial modulator in the tumorigenesis and malignant progression of GBM [72]. Sun et al. [24] revealed that activation of solute carrier family 10 member 3 (SLC10A3) induces nuclear phosphorylation of STAT3. Subsequently, the phosphorylated STAT3 binds to the promoter of GPX4. Through this ferroptosis-suppressive mechanism, STAT3 enhances GBM cell survival and contributes to therapy resistance under oxidative stress conditions. This molecular interaction results in the upregulation of GPX4 expression, thereby attenuating ferroptosis. These findings underscore the SLC10A3-STAT3-GPX4 axis as a promising therapeutic target for GBM.

### 4.2. Molecular Mechanism of Ferroptosis in NB

**Figure 3:**
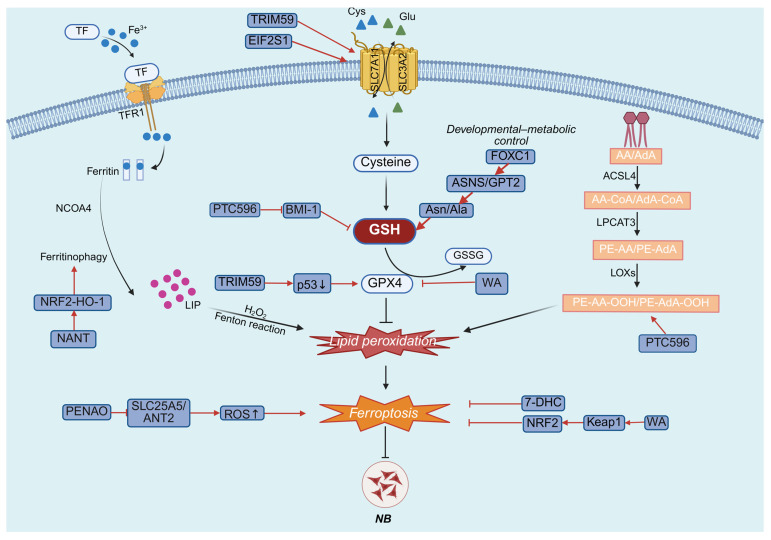
NB-specific metabolic and transcriptional control of ferroptosis. In NB, ferroptosis can be modulated through diverse mechanisms, including alterations in lipid metabolism, mitochondrial metabolism, and iron metabolism, with GSH homeostasis functioning as a central regulatory hub coordinating these processes. Abbreviations: TRIM59, tripartite motif 59; EIF2S1, eukaryotic translation initiation factor 2 subunit alpha; PTC596, unesbulin; BMI-1, B-cell-specific Moloney murine leukemia virus insertion site 1; Asn, asparagine; Ala, alanine; ASNS, asparagine synthetase; GPT2, glutamic-pyruvic transaminase 2; FOXC1, forkhead box C1; NANT, N-acetylneuraminyltransferase; PENAO, penaoquinone; ANT2, adenine nucleotide translocase-2; ROS, reactive oxygen species; 7-DHC, 7-Dehydrocholesterol; WA, withaferin A; Keap1, kelch-like ECH-associated protein 1; NRF2, erythroid-derived 2; SLC25A5, solute carrier family 25 member 5; p53, protein 53.

Unlike GBM, ferroptosis regulation in NB is embedded within a pediatric developmental context and is strongly influenced by oncogenic amplification and metabolic vulnerability. In NB, ferroptosis is intricately regulated by the interplay among GSH homeostasis, lipid metabolic remodeling, intracellular iron dynamics, and mitochondrial redox function (Figure 3).

#### 4.2.1. GPX4/GSH Steady-State System

Tripartite motif-containing protein 59 (TRIM59) interacts directly with and mediates ubiquitin-dependent degradation of the tumor suppressor p53, leading to upregulation of ferroptosis-inhibitory proteins GPX4 and SLC7A11. Through this mechanism, the TRIM59–p53 axis sustains the GPX4/GSH redox buffer, thereby suppressing ferroptosis and promoting NB progression as well as chemoresistance [58].

As a cystine transporter, SLC7A11 is a key molecule for GSH biosynthesis. GPX4, on the other hand, depends on GSH to carry out its function of scavenging lipid ROS. Li et al. [59] have clearly elucidated that forkhead box C1 (FOXC1) promotes the biosynthesis of asparagine (Asn) and alanine (Ala) by upregulating the expression of asparagine synthetase (ASNS) and glutamic-pyruvic transaminase 2 (GPT2). This metabolic reprogramming further enhances the cystathionine β-synthase (CBS)-mediated transsulfuration pathway, leading to increased GSH biosynthesis. Consequently, GPX4 is activated to scavenge lipid ROS.

PTC596 suppresses the stemness-associated gene BMI-1, significantly decreasing intracellular GSH levels and elevating lipid peroxidation, thereby inducing ferroptotic cell death in NB without altering GPX4 enzymatic activity [60].

MYCN, a member of the MYC family encoding a basic helix-loop-helix transcription factor, is frequently amplified in high-risk neuroblastoma and is associated with aggressive disease and poor prognosis, and also plays a critical role in modulating ferroptosis. MYCN-amplified NB cells display strong dependency on cysteine and exhibit heightened vulnerability to ferroptosis upon cysteine deprivation. Decreased cysteine availability forces its metabolic allocation toward protein synthesis rather than GSH production, resulting in compromised GPX4 activity and heightened lipid peroxidation. This mechanism provides a potential explanation for spontaneous tumor regression observed in subsets of low-risk NB. Notably, pharmacological inhibition of cystine uptake and the transsulfuration pathway, combined with GPX4 inhibition, effectively induced tumor regression in MYCN-amplified orthotopic NB models [25]. Hassannia et al. provided evidence demonstrating that withaferin A (WA) exhibits dose-dependent dual effects on ferroptosis induction. Specifically, WA can either activate the NRF2 pathway by targeting Kelch-like ECH-associated protein 1 (Keap1), which represents non-canonical ferroptosis induction, or inactivate GPX4, a key player in canonical ferroptosis induction [65].

#### 4.2.2. Iron Homeostasis

Xue et al. [73] demonstrated that N-acetylneuraminyltransferase (NANT) activates the NRF2–HO-1 signaling axis, promoting heme degradation and releasing Fe^2+^. Concurrently, NANT enhances ferritinophagy, resulting in liberation of ferritin-sequestered iron. The combined surge in intracellular Fe^2+^ amplifies Fenton-driven lipid peroxidation and triggers ferroptosis.

#### 4.2.3. Lipid Reprogramming

Malignant cells exhibit a critical reliance on lipid metabolic pathways. These pathways not only support adenosine triphosphate (ATP) generation but also maintain membrane homeostasis and supply biosynthetic precursors for lipid-derived secondary messengers [74].

7-Dehydrocholesterol (7-DHC), an intermediate metabolite in the cholesterol biosynthetic pathway, possesses substantially higher peroxyl radical reactivity compared to PUFAs. This distinctive property enables it to be preferentially oxidized, thereby shielding membrane phospholipids from peroxidation and cleavage into membrane-destructive truncated products [64]. Consequently, 7-DHC mitigates ferroptosis, enhances tumor cell chemoresistance, and promotes tumor cell invasion and metastasis.

#### 4.2.4. Mitochondrial Dynamics

Research has shown that solute carrier family 25 member 5/adenine nucleotide translocase 2 (SLC25A5/ANT2) is highly expressed in NB, and this overexpression is closely correlated with a poor prognosis in patients. Penaoquinone (PENAO) specifically targets SLC25A5/ANT2 to disrupt the translocation of mitochondrial ATP, leading to the accumulation of ROS. The elevated ROS levels induced by the combination therapy of suberoylanilide hydroxamic acid (SAHA) and PENAO may activate ferroptosis-related signaling pathways. Notably, PENAO increases intracellular GSH levels, while SAHA decreases GSH content. This shift in redox homeostasis is likely to modulate the sensitivity of NB cells to ferroptosis [66]. Together, these mechanisms indicate that ferroptosis regulation in NB is closely coupled to developmental programs, oncogenic MYCN signaling, and metabolic dependencies.

## 5. The Role of Ferroptosis in the Diagnosis of GBM and NB

In this section, we summarize the ferroptosis-related biomarkers that have diagnostic or prognostic value, which are crucial for improving clinical decision-making [75], patient stratification [76], and personalized treatment (Table 2).

### 5.1. The Role in the Diagnosis of GBM

Currently, there is a lack of effective therapeutic approaches and robust prognostic biomarkers for predicting treatment responses and clinical outcomes in GBM. Moreover, the inherent heterogeneity of GBM tumors hinders the clinical translation of candidate molecular markers [87]. Therefore, the discovery of precise ferroptosis-associated biomarkers represents a promising direction for addressing these unmet needs.

Integrative bioinformatics analysis identified three ferroptosis-suppressive differentially expressed genes (FS-DEGs), namely, CD44 (cluster of Differentiation 44), heat shock protein family B (small) member 1 (HSPB1), and SLC40A1 could potentially serve as valuable prognostic and diagnostic biomarkers for GBM patients. Their expression is closely associated with immunosuppression in the tumor microenvironment [77]. Lv et al. [78] reported that microfibril-associated protein 4 (MFAP4) functions as a ferroptosis-related biomarker strongly associated with adverse clinical features, immune cell infiltration, and resistance to immunotherapy. Mechanistically, MFAP4 was linked to the regulation of several ferroptosis-related genes, including PTGS2, GPX4, and SLC40A1, and promoted GBM cell proliferation, migration, and invasion . Wu et al. [79] established a 5-gene ferroptosis-derived signature through multi-omics screening of The Cancer Genome Atlas-GBM (TCGA-GBM) and Chinese Glioma Genome Atlas-GBM (CGGA-GBM) cohorts. The resulting risk score independently stratifies newly diagnosed GBM patients into high- and low-risk groups with distinct overall survival probabilities, providing a minimally invasive transcriptional metric for prognostic stratification of GBM.

Preclinical studies by Liu et al. demonstrated that ferroptosis inducers, when combined with ICB therapies, elicit a robust synergistic therapeutic efficacy in murine models of GBM [88]. Feng et al. [89] demonstrated that growth differentiation factor 15 (GDF15), a stress-responsive cytokine highly expressed in recurrent and radioresistant GBM, not only suppresses radiation-induced ferroptosis but also modulates the intratumoral immune landscape. Mechanistically, GDF15 stabilizes NRF2 by inhibiting ubiquitin-mediated degradation, thereby upregulating ferroptosis-suppressive genes and reducing lipid peroxidation to promote GBM cell survival under radiotherapy. Concurrently, GDF15 facilitates the recruitment and polarization of M2 macrophages and enhances Treg infiltration, fostering an immunosuppressive TME that further reinforces radio resistance. Clinically, overexpression of GDF15 in GBM tissues is correlated with an adverse prognosis, a higher radiation sensitivity index (RSI), and an increased M2/M1 macrophage ratio, as validated by transcriptomic analysis of public datasets and clinical specimens. Thus, GDF15 may serve as a potential prognostic biomarker for GBM. Fu et al. [80] identified the long non-coding RNA PELATON (LINC01272) as a ferroptosis-associated biomarker that is significantly upregulated in glioblastoma tissues and correlates with unfavorable clinical outcomes. Incorporation of PELATON into a ferroptosis-related prognostic signature enabled effective risk stratification of GBM patients, supporting the potential utility of ferroptosis-linked lncRNAs as diagnostic and prognostic biomarkers.

### 5.2. The Role in the Diagnosis of NB

NB is a challenging pediatric malignancy. While MYCN amplification, detected in 16–25% of NB cases, stands as one of the most powerful adverse prognostic biomarkers, risk stratification for NB patients lacking this genetic alteration remains considerably more intricate [90,91].

Translational research by Tan et al. revealed that a ferroptosis-related gene signature comprising leukemia inhibitory factor receptor (LIFR), tumor protein p53 (TP53), neuroblastoma RAS viral oncogene homolog (NRAS), and oxysterol binding protein like 9 (OSBPL9) not only modulates ferroptosis sensitivity in MYCN-amplified NB cells but also functions as a robust independent prognostic indicator for patients with this high-risk subtype [84].

The m^6^A and ferroptosis-related prognostic index (MFPI), constructed using 19 key genes including aldo-keto reductase family 1 member C1 (AKR1C1), interferon alpha 10 (IFNA10), cysteine dioxygenase type 1 (CDO1), heterogeneous nuclear ribonucleoprotein A2/B1 (HNRNPA2B1), and FTH1 has been validated to accurately predict overall survival (OS) in NB patients, with superior performance in both MYCN-amplified and non-amplified subtypes [85]. Similarly, Chen et al. developed an eight-gene ferroptosis-related signature including prominin 2 (PROM2), aurora kinase A (AURKA), and STAT3 that robustly stratifies NB patients into risk groups, exhibiting strong predictive performance across validation cohorts [83]. They propose a novel strategy for risk stratification of NB patients using this FRG-based signature, which exhibits robust predictive performance in both training and validation cohorts. Moreover, they found that the high-risk group identified by this signature was associated with more aggressive clinical phenotypes and was positively correlated with the enrichment of immune-related biological processes and infiltrating immune cells. Additionally, these eight FRGs were linked to key ferroptosis regulatory pathways and immune checkpoints. Specifically, AURKA was highlighted as a potential independent prognostic biomarker for NB due to its distinct high expression in high-risk subgroups [83].

In a translational study focusing on high-risk NB, Cheng et al. [86] identified two ferroptosis-related hub genes, MYCN and ribonucleotide reductase regulatory subunit M2 (RRM2), along with multiple FRGs, to develop a prognostic signature and a machine-learning-based recurrence model for NB patients. Notably, the high-risk group stratified by this model exhibited significantly worse OS and event-free survival (EFS), and was positively correlated with aggressive phenotypes and enrichment of tumor-infiltrating immune cells. Additionally, RRM2, a key regulator of DNA synthesis and redox homeostasis, was emphasized as a critical FRG that synergizes with MYCN to drive NB progression, highlighting its potential as an independent prognostic biomarker and therapeutic target for high-risk NB.

## 6. Ferroptosis as a Therapeutic Vulnerability in GBM and NB

In this section, we summarize ferroptosis-based therapeutic strategies and highlight their potential for overcoming treatment resistance in nervous system malignancies (Table 3).

### 6.1. GBM: Ferroptosis Regulation Shaped by Metabolic Plasticity and Therapy Resistance

In GBM, ferroptosis is tightly linked to metabolic plasticity, redox adaptation, and resistance to standard therapies, positioning it as a context-dependent therapeutic vulnerability. Despite multimodal therapy, GBM remains highly refractory, motivating exploration of ferroptosis-based strategies as adjunctive interventions [104,105].

Tan et al. [92] demonstrated that 15,16-dihydrotanshinone I (DHI), a natural component extracted from Salvia miltiorrhiza Bunge, significantly enhances the susceptibility of GBM cells to ferroptosis. This effect is mediated by GPX4 downregulation and ACSL4 upregulation, which promotes lipid peroxidation and mitochondrial membrane potential collapse, ultimately inducing GBM cell death. This finding indicates that DHI may hold promise as a therapeutic agent for GBM via targeting ferroptosis. Lu et al. [93] demonstrated that brucine induces ferroptosis in GBM cells via endoplasmic reticulum stress-mediated activation of activating transcription factor 3 (ATF3). ATF3 promotes hydrogen peroxide accumulation and iron overload by upregulating NOX4/SOD1 and suppressing the xc^−^/catalase axis, thereby amplifying lipid peroxidation and ferroptotic cell death, highlighting ATF3 as a potential ferroptosis-related therapeutic target in GBM.

Chemoresistance—particularly resistance to temozolomide (TMZ)—remains a major clinical challenge in GBM management. Yang et al. [106] identified polymerase I and transcript release factor (PTRF/Cavin-1) as a prognostic biomarker that drives TMZ resistance by enhancing extracellular vesicle (EV)-mediated TMZ efflux. This decreases intracellular drug concentration and suppresses ferroptosis-related cell death. Importantly, sequential treatment with TMZ followed by chloroquine (CQ) effectively reverses resistance by inhibiting PTRF-dependent EV secretion. This discovery suggests new opportunities for ferroptosis-centered combination therapy in overcoming chemoresistance.

The ferroptosis-regulating agent albumin-bound paclitaxel (ABX) may synergize with TMZ by disrupting DNA repair pathways (involving XPC/ERCC1) and modulating HO-1/GPX4 signaling pathway. This combination strategy holds substantial potential for overcoming TMZ resistance in GBM [107]. Given that a major limitation of current ferroptosis inducers is blood–brain barrier (BBB) penetration, biomimetic nano-delivery systems have emerged as critical enablers [108]. Cao et al. engineered Angiopep-2-modified macrophage membrane-camouflaged nanoparticles (Ang-MMsaNPs) for the targeted delivery of arachidonate 15–lipoxygenase small activating RNA (saALOX15) to GBM cells [109]. Ang-MMsaNPs enable BBB penetration and targeted delivery of saALOX15, promoting ALOX15-dependent lipid peroxidation and ferroptotic cell death in GBM. Notably, ALOX15 activation synergizes with radiotherapy to suppress tumor progression and overcome treatment resistance.

The potassium channel gene KCNA1 plays an important role in GBM ferroptosis susceptibility. KCNA1 deficiency increases mitochondrial damage, intracellular ROS production, and lipid peroxidation by downregulating the SLC7A11/GPX4 axis [99]. In orthotopic xenograft models, KCNA1 loss inhibited tumor proliferation and invasion and improved survival outcomes. These findings highlight KCNA1 as a promising therapeutic target for ferroptosis-based GBM therapy.

### 6.2. NB: Developmental and MYCN-Driven Ferroptosis Dependencies

In neuroblastoma, ferroptosis induction exploits unique vulnerabilities linked to developmental metabolism and MYCN-driven redox dependency, highlighting therapeutic distinctions from both adult brain tumors and non-neural cancers [110]. In NB, ferroptotic sensitivity is strongly influenced by developmental origin and oncogenic drivers such as MYCN, resulting in ferroptosis regulatory networks distinct from those observed in adult brain tumors [111]. Ferroptosis in NB can be triggered by small molecules such as erastin, sulfasalazine, and sorafenib, or by inhibiting the GPX4/xCT antioxidant axis. Sensitization to ferroptosis can be achieved via ferroportin knockdown, MYCN amplification-related TFR1 upregulation, protein kinase C alpha (PKCα) inhibition, or FTH1 deficiency [112]. Transcription factor 4 (TCF4) has the potential to inhibit ferroptosis in NB cells through the TCF4-GPX4 axis by directly transactivating GPX4 transcription. Moreover, elevated expression of TCF4 is strongly associated with enhanced proliferation and malignant progression in NB. Therefore, targeting the TCF4-GPX4 axis represents a promising approach for the treatment of high-risk NB [101].

Numerous compounds have been identified in recent studies that can inhibit NB progression via ferroptosis modulation. For instance, Shir et al. [103] reported that regorafenib, a multi-kinase inhibitor approved by the Food and Drug Administration (FDA), specifically targets dihydroorotate dehydrogenase (DHODH), a key enzyme in de novo pyrimidine synthesis and a prognostic marker for high-risk NB. Regorafenib synergistically modulates the mevalonate pathway, inducing ferroptosis in NB cells and patient-derived organoids. Furthermore, pharmacological treatment with pomiferin, a prenylated isoflavonoid isolated from Maclura pomifera, effectively inhibits cell proliferation, induces ferroptosis and apoptosis, and synergizes with conventional chemotherapeutics in NB cells [113].

The iron chelator VLX600 represents a translationally advanced strategy that targets mitochondrial metabolism. By inhibiting electron transport chain (ETC) complexes, VLX600 suppresses oxidative phosphorylation and downregulates MYCN via the mTOR pathway [114]. Notably, this agent effectively induces cell death in 3D tumor models independent of MYCN amplification status, offering a metabolic bypass to conventional drug resistance in high-risk NB [115,116].

Ferroportin (Fpn), the sole known iron exporter responsible for regulating intracellular iron levels, enhances erastin-induced ferroptosis through knockdown-mediated iron-dependent lipid ROS accumulation, positioning it as a viable therapeutic target for NB [29,117]. Conversely, mitochondrial ferritin (FtMt), a mitochondrial iron-storage protein, confers protection against erastin-triggered ferroptosis, highlighting its regulatory role in mitochondrial iron homeostasis [118]. Additionally, downregulation of cell division cycle 27 (CDC27) decreases MYCN-regulated ornithine decarboxylase 1 (ODC1) expression, thereby attenuating CDC27-driven tumor-promoting activity in NB cells [63].

## 7. Clinical Challenges in Targeting Ferroptosis with Therapeutic Agents

Ferroptosis-inducing agents (FINs) can synergize with conventional therapies and immunotherapy in preclinical models [12]. Nonetheless, clinical translation is hindered by several critical challenges.

First, the intricate, multilayered regulatory network governing ferroptosis and its context-dependent paradoxical functions significantly impede clinical application. Although ferroptosis suppresses malignant cells, under certain metabolic or microenvironmental conditions it may inadvertently promote tumorigenesis [119]. Therefore, rigorous mechanistic studies are essential to delineate the conditions under which ferroptosis acts as a tumor suppressor versus a tumor promoter.

Second, ferroptosis resistance is a major barrier limiting the therapeutic efficacy of FINs, despite encouraging preclinical outcomes [120]. Tumor cells can acquire resistance through activation of adaptive antioxidant systems, metabolic reprogramming, remodeling of the TME to enhance redox buffering, and tumor-intrinsic genetic alterations [121,122,123]. These resistance mechanisms underscore the need for rational combinatorial strategies and biomarker-guided patient stratification.

Third, translating ferroptosis modulators into clinical practice faces pivotal challenges, particularly in terms of pharmacokinetics, safety, and drug delivery [124]. Critical pharmacokinetic hurdles include poor solubility and bioavailability, which limit systemic efficacy [125]. There are also issues such as inadequate tissue penetration, for example, limited blood–brain barrier crossing in central nervous system disorders, metabolic instability leading to rapid degradation, short elimination half-lives requiring frequent dosing, and suboptimal physicochemical properties complicating absorption, distribution, metabolism, and excretion (ADME) profiles [126,127].

Fourth, direct intravenous administration of many small-molecule ferroptosis inducers may elicit severe systemic toxicity [128,129]. Compared with conventional drug delivery approaches, nanotechnology-based nano-delivery systems represent a highly promising platform for ferroptosis-targeted therapy. They feature controllable drug release and reduced off-target toxicity [130,131].

To address these critical questions, future research should focus on several key avenues. First, deciphering the molecular crosstalk between ferroptosis and other regulated cell death pathways (e.g., apoptosis, necroptosis, autophagy) is essential to map interconnected signaling networks and integrate these events into a unified cellular death regulatory framework. Second, investigating the regulatory roles of non-coding RNAs (ncRNAs), including miRNAs, lncRNAs, and circRNAs, in ferroptotic signaling cascades will uncover novel molecular targets and mechanisms to modulate ferroptosis with high specificity. Third, optimizing rational combination therapeutic strategies—such as ferroptosis inducers combined with immunotherapy, radiotherapy, targeted agents (e.g., EGFR inhibitors, AR antagonists), or nano-delivery systems—is imperative. This will enhance anti-tumor efficacy, overcome drug resistance, and mitigate off-target toxicity, with a focus on clarifying synergistic molecular mechanisms and identifying predictive biomarkers for patient stratification.

## 8. Discussion

Recent advances have substantially expanded our understanding of ferroptosis as a regulated cell death with therapeutic relevance in cancer [22]. Building on prior reviews, the present work emphasizes that ferroptosis regulation in nervous system tumors is profoundly shaped by developmental context, metabolic wiring, and tumor microenvironmental constraints. Through a comparative analysis of glioblastoma and neuroblastoma, we illustrate that ferroptosis is not a uniform vulnerability, but rather a context-conditioned process whose regulatory logic and therapeutic implications differ markedly between adult and pediatric malignancies [132,133].

In GBM, ferroptosis-based strategies are constrained by blood–brain barrier permeability, pronounced intratumoral heterogeneity, and robust antioxidant buffering [134], often necessitating targeted or combinatorial approaches to overcome adaptive resistance. In contrast, ferroptotic susceptibility in neuroblastoma is closely linked to developmental stage and oncogenic drivers such as MYCN, which reprogram cysteine metabolism and redox homeostasis, creating distinct therapeutic vulnerabilities. Importantly, the intrinsic sensitivity of normal neural tissues to lipid peroxidation and iron imbalance further underscores the need for precise and tumor-specific modulation of ferroptosis in nervous system malignancies [58].

Some key approaches targeting ferroptosis have already been developed, significantly expanding their potential applications in cancer treatment Ferroptosis also intersects with anti-tumor immunity, suggesting opportunities for rational combination with immunotherapy [135].

Ferroptosis induction within tumor cells holds the potential to abrogate chemotherapy and radiotherapy resistance in malignant cells. For example, Wei et al., [136] reported that knockdown of FXR1 could overcome TMZ resistance by promoting ferroptosis. Notably, ex vivo human glioma slice cultures treated with the SLC7A11 inhibitor IKE plus radiotherapy exhibited increased ROS levels relative to monotherapy [137], providing novel insights into the combination of ferroptosis inducers and chemotherapy.

Despite encouraging preclinical progress, several challenges continue to limit clinical translation, including incomplete mechanistic understanding, tumor heterogeneity, and the scarcity of robust in vivo and clinical validation models. Future efforts should prioritize integrative multi-omics analyses, physiologically relevant model systems, and rational combination strategies to define predictive biomarkers and safely harness ferroptosis for therapeutic benefit [119,138,139]. In conclusion, this review highlights ferroptosis as a promising yet context-dependent therapeutic avenue in nervous system tumors. Advancing a nuanced, tumor-specific understanding of ferroptosis regulation will be essential for translating ferroptosis-targeted strategies into precision oncology and improving outcomes for patients with these devastating malignancies.

## Figures and Tables

**Table 1:** The molecular mechanism of ferroptosis in malignant tumors of the nervous system.

Tumor	Drug	Target	Ferroptosis (Inducer Inhibitor)	Mechanism	Model	Reference
GBM		IFI16	Inhibit	Activate HMOX1 and upregulate GPX4	In vitro and in vivo	[42]
	Disulfiram		Induce	Downregulate the expression of xCT and GPX4, at the same time enhance the ROS level	In vitro	[43]
	ALZ003		Induce	Downregulate the expression of GPX4 and lead to the ROS accumulation	In vitro and in vivo	[44]
		SIRT3	Inhibit	Maintain mitochondrial homeostasis and regulate SLC7A11	In vitro and in vivo	[45]
	Fatostatin		Induce	Inhibit the AKT/mTORC1/GPX4 signaling pathway	In vitro and in vivo	[46]
		ZDHHC8	Inhibit	Mediate SLC7A11 S-palmitoylation	In vitro and in vivo	[47]
		C5aR1	Inhibit	Promote METTL3-dependent GPX4 expression through ERK1/2	In vitro and in vivo	[48]
		TRAF3	Induce	Inhibit ECH1-mediated oxidation of PUFA	In vitro and in vivo	[49]
		CircMAN1A2	Induce	Bind TEP1, block TEP1-KEAP1 interaction, and promote NRF2 degradation	In vitro and in vivo	[50]
		IDO1	Inhibit	Regulate FTO-mediated m6A methylation and SLC7A11 mRNA stability	In vitro	[51]
	PLB		Induce	Increase in MDA and ROS levels, reduce of GSH levels and downregulate of xCT and GPX4 expressions	In vitro and in vivo	[52]
		lncRNA LINC01088	Inhibit	Upregulate SLC7A11 expression by stabilizing HLTF via USP7-mediated deubiquitination	In vitro and in vivo	[53]
	Procyanidin B1		Induce	Promote NRF2 degradation via ubiquitination mediated by NRF2-PSMC3 interaction, leading to H_2_O_2_ accumulation	In vitro and in vivo	[54]
		S670	Induce	Generate ROS and inhibit STX17-mediated fusion of autophagosome and lysosome	In vitro and in vivo	[55]
		SLC10A3	Inhibit	STAT3-mediated GPX4 upregulation	In vitro and in vivo	[24]
		HSP27	Inhibit	Fe^2+^↓/ROS↓/Mitochondrial membrane potential↑	In vitro and in vivo	[56]
	Orexin-A		Induce	NFE2L2↓-TFRC↑/GPX4↓ axis	In vitro and in vivo	[57]
NB		TRIM59	Inhibit	Bind to and ubiquitinate p53; upregulate GPX4 and SLC7A11	In vitro and in vivo	[58]
		FOXC1	Inhibit	Enhance GSH synthesis; activate GPX4	In vitro and in vivo	[59]
		PTC-596	Induce	Reduce GSH; induce lipid peroxidation	In vitro	[60]
		EIF2S1	Inhibit	GPX4/SLC7A11 axis	In vitro and in vivo	[61]
		PRDX6	Inhibit	PRDX6 and SCLY act in parallel to facilitate the decomposition of Sec into HSe^−^, which supplies SEPHS2 for the biosynthesis of selenophosphate and sustains GPX4 activity	In vitro and in vivo	[62]
		CDC-27	Induce	Upregulate ODC1 expression	In vitro and in vivo	[63]
	7-DHC		Inhibit	Act as a preferred peroxyl radical trap, inhibit phospholipid oxidation	In vitro and in vivo	[64]
	WA		Induce/ Inhibit	Activate the NRF2 pathway by targeting Kelch-like ECH-associated protein 1(Low dose); inactivate GPX4 (High dose)	In vitro and in vivo	[65]
	SAHA	PENAO	Induce	Target SLC25A5 to disrupt mitochondrial ATP transport; induce ROS accumulation	In vitro and in vivo	[66]

Abbreviations: 7-DHC, 7-Dehydrocholesterol; C5aR1, C5a receptor 1; CDC-27, cell division Cycle 27; ECH1, enoyl-CoA hydratase 1; EIF2S1, EIF-2alpha; FTO, fat mass and obesity-associated protein; FOXC1, forkhead box C1; GBM, glioblastoma; GPX4, Glutathione Peroxidase 4; GSH, glutathione; HLTF, Helicase-like Transcription Factor; HMOX1, heme oxygenase 1 gene; HSP27, Heat shock protein 27; IDO1, indoleamine 2,3-dioxygenase 1; IFI16, interferon γ-inducible protein 16; KEAP1, kelch-like ECH-associated protein 1; MDA, Malondialdehyde; METTL3, methyltransferase like 3; NB, neuroblastoma; NFE2L2, NFE2 like bZIP transcription factor 2; NRF2, erythroid-derived 2; ODC1, ornithine decarboxylase 1; PENAO, penaoquinone; PLB, Phospholipase B; PRDX6, peroxiredoxin 6; PSMC3, proteasome 26S subunit; PUFA, Polyunsaturated fatty acid; ROS, reactive oxygen species; SAHA, suberoylanilide hydroxamic acid; SCLY, selenocysteine lyase; SEPHS2, selenophosphate synthetase 2; SIRT3, sirtuin 3; SLC7A11, solute carrier family 7 member 11; SLC10A3, solute carrier family 10 member 3; SLC25A5, solute carrier family 25 member 5; STAT3, Signal transducer and activator of transcription 3; STX17, syntaxin 17; TEP1, telomerase associated protein 1; TFRC, transferrin receptor; TRAF3, TNF receptor-associated factor 3; TRIM59, tripartite motif containing 59; USP7, ubiquitin-specific protease 7; WA, withaferin A; ZDHHC8, Zinc finger DHHC-type containing 8.

**Table 2:** The roles of ferroptosis in the diagnosis of nervous system tumors.

Tumor	Target	Relationship with Ferroptosis	Biological Function	Reference
GBM	CD44, HSPB1 and SLC40A1	Ferroptosis and immune-related differentially expressed genes	Worse OS	[77]
	MFAP4	Ferroptosis and immune-related differentially expressed gene	Worse OS	[78]
	OSMR, G0S2, IGFBP6, IGHG2, and FMOD	Ferroptosis and immune-related differentially expressed genes	Worse OS	[79]
	LncRNA PELATON	Ferroptosis-related lncRNA	Worse OS	[80]
	ACSL3, ACSL4 and ALOX5	Ferroptosis and lipid metabolism-related genes	Better OS	[81]
	STEAP3	Ferroptosis-related gene	Worse OS	[82]
NB	PROM2, ULK2, STAT3	Ferroptosis-related genes	Better OS	[83]
	TP53, NRAS	Ferroptosis-related genes	Worse OS	[84]
	AKR1C1	Ferroptosis and lipid metabolism-related gene	Worse OS	[85]
	RRM2	Ferroptosis and DNA repair-related gene	Worse OS	[86]

Abbreviations: ACSL3, acyl-CoA synthetase long-chain family member 3; ACSL4, acyl-CoA synthetase long-chain family member 4; AKR1C1, aldo-keto reductase family 1 member C1; ALOX5, arachidonate 5-lipoxygenase; CD44, cluster of differentiation 44; FMOD, fibromodulin; G0S2, G0/G1 switch 2; GBM, glioblastoma; HSPB1, heat shock protein family B (small) member 1; IGFBP6, insulin-like growth factor binding protein 6; IGHG2, immunoglobulin heavy constant gamma 2; MFAP4, microfibril-associated protein 4; NB, neuroblastoma; NRAS, neuroblastoma RAS viral oncogene homolog; PROM2, prominin 2; RRM2, ribonucleotide reductase regulatory subunit M2; SLC40A1, solute carrier family 40 member 1; STAT3, signal transducer and activator of transcription 3; STEAP3, six-transmembrane epithelial antigen of prostate 3; ULK2, unc-51 like autophagy activating kinase 2.

**Table 3:** The roles of ferroptosis in the treatment of nervous system malignancies.

Tumor	Drug	Target	Ferroptosis (Inducer Inhibitor)	Biological Function	Model	Reference
GBM	DHI		Induce	Suppress cell proliferation, augment cytotoxicity and cellular damage and impair mitochondrial function	In vitro	[92]
		ATF3	Induce	Suppress clonogenicity, induce H_2_O_2_ accumulation and provoke iron overload	In vitro and in vivo	[93]
		EGFR	Induce	Downregulate global m^6^A levels, mediate ALKBH5 nuclear localization and promote GSH biosynthesis	In vitro and in vivo	[94]
	Cannabidiol		Induce	Trigger autophagy and reduce mitochondrial membrane potential	In vitro	[95]
		APOC1	Inhibit	Enhance migration, invasion, and 3D growth; maintain cellular redox homeostasis	In vitro and in vivo	[96]
	DHA		Induce	Inhibit cell proliferation and clonogenicity, elicit endoplasmic reticulum stress and trigger lipid peroxidation	In vitro and in vivo	[97]
		COPZ1	Inhibit	Promote cell proliferation and clonogenicity and facilitate in vivo tumor growth	In vitro and in vivo	[98]
		KCNA1	Inhibit	Upregulate EMT; enhance migration and invasion	In vitro and in vivo	[99]
		CENPA	Inhibit	Regulate GSCs stemness and proliferation	In vitro and in vivo	[100]
NB		TCF4	Inhibit	Suppress ferroptosis of NB cell	In vitro and in vivo	[101]
		EIF2S1	Inhibit	Promote cell proliferation, migration, and invasion; accelerate tumor growth	In vitro and in vivo	[61]
	BHT		Inhibit	Reduce cellular oxidative potential and block lipid peroxidation	In vitro and in vivo	[102]
	Regorafenib	DHODH	Inhibit	Maintain the mevalonate pathway	In vitro	[103]
		OGT-FOXC1	Inhibit	Activate asparagine/alanine synthesis; promote NB cell proliferation, invasion, and in vivo tumor growth and metastasis	In vitro and in vivo	[59]

Abbreviations: ALKBH5, alkB homolog 5; APOC1, apolipoprotein C1; ATF3, activating transcription factor 3; BHT, butylated hydroxytoluene; CENPA, centromere protein A; COPZ1, coatomer protein complex subunit zeta 1; DHA, docosahexaenoic acid; DHI, 15,16-Dihydrotanshinone I; DHODH, dihydroorotate dehydrogenase; EGFR, epidermal growth factor receptor; EIF2S1, eukaryotic translation initiation factor 2 subunit alpha; EMT, epithelial–mesenchymal transition; GBM, glioblastoma; GSCs, glioma stem cells; GSH, glutathione; KCNA1, potassium voltage-gated channel subfamily A member 1; NB, neuroblastoma; OGT-FOXC1, O-GlcNAc transferase–forkhead box C1 regulatory axis; TCF4, transcription factor 4.

## Data Availability

No new data were created or analyzed in this study. Data sharing is not applicable to this article.

## References

[1] Schaff L.R., Mellinghoff I.K. (2023). Glioblastoma and Other Primary Brain Malignancies in Adults: A Review. JAMA.

[2] Rong L., Li N., Zhang Z. (2022). Emerging therapies for glioblastoma: Current state and future directions. J. Exp. Clin. Cancer Res..

[3] Singh S., Dey D., Barik D., Mohapatra I., Kim S., Sharma M., Prasad S., Wang P., Singh A., Singh G. (2025). Glioblastoma at the crossroads: Current understanding and future therapeutic horizons. Signal Transduct. Target. Ther..

[4] Foreman K.J., Marquez N., Dolgert A., Fukutaki K., Fullman N., McGaughey M., Pletcher M.A., Smith A.E., Tang K., Yuan C.W. (2018). Forecasting life expectancy, years of life lost, and all-cause and cause-specific mortality for 250 causes of death: Reference and alternative scenarios for 2016-40 for 195 countries and territories. Lancet.

[5] Ostrom Q.T., Price M., Neff C., Cioffi G., Waite K.A., Kruchko C., Barnholtz-Sloan J.S. (2023). CBTRUS Statistical Report: Primary Brain and Other Central Nervous System Tumors Diagnosed in the United States in 2016–2020. Neuro Oncol..

[6] Lan Z., Li X., Zhang X. (2024). Glioblastoma: An Update in Pathology, Molecular Mechanisms and Biomarkers. Int. J. Mol. Sci..

[7] Ismailov A., Spallone A., Belogurov A., Herbert A., Poptsova M. (2025). Molecular biology of the deadliest cancer—Glioblastoma: What do we know?. Front. Immunol..

[8] Ramachandran M., Yu D., Dyczynski M., Baskaran S., Zhang L., Lulla A., Lulla V., Saul S., Nelander S., Dimberg A. (2017). Safe and Effective Treatment of Experimental Neuroblastoma and Glioblastoma Using Systemically Delivered Triple MicroRNA-Detargeted Oncolytic Semliki Forest Virus. Clin. Cancer Res..

[9] Nakagawara A., Li Y., Izumi H., Muramori K., Inada H., Nishi M. (2018). Neuroblastoma. Jpn. J. Clin. Oncol..

[10] Zafar A., Wang W., Liu G., Wang X., Xian W., McKeon F., Foster J., Zhou J., Zhang R. (2021). Molecular targeting therapies for neuroblastoma: Progress and challenges. Med. Res. Rev..

[11] Dixon S.J., Olzmann J.A. (2024). The cell biology of ferroptosis. Nat. Rev. Mol. Cell Biol..

[12] Mou Y., Wang J., Wu J., He D., Zhang C., Duan C., Li B. (2019). Ferroptosis, a new form of cell death: Opportunities and challenges in cancer. J. Hematol. Oncol..

[13] Chen T., Leng J., Tan J., Zhao Y., Xie S., Zhao S., Yan X., Zhu L., Luo J., Kong L. (2023). Discovery of Novel Potent Covalent Glutathione Peroxidase 4 Inhibitors as Highly Selective Ferroptosis Inducers for the Treatment of Triple-Negative Breast Cancer. J. Med. Chem..

[14] Zhang Y., Guo R., Li J., Zhu L. (2022). Research progress on the occurrence and therapeutic mechanism of ferroptosis in NSCLC. Naunyn Schmiedebergs Arch. Pharmacol..

[15] Li C., Yin X., Liu Z., Wang J. (2022). Emerging Potential Mechanism and Therapeutic Target of Ferroptosis in PDAC: A Promising Future. Int. J. Mol. Sci..

[16] Ajoolabady A., Tang D., Kroemer G., Ren J. (2023). Ferroptosis in hepatocellular carcinoma: Mechanisms and targeted therapy. Br. J. Cancer.

[17] Xu L., Liu Y., Chen X., Zhong H., Wang Y. (2023). Ferroptosis in life: To be or not to be. Biomed. Pharmacother..

[18] Wang H., Liu Y., Che S., Li X., Tang D., Lv S., Zhao H. (2024). Deciphering the link: Ferroptosis and its role in glioma. Front. Immunol..

[19] Mashayekhi S., Majedi H., Dehpour A.R., Dehghan S., Jafarian M., Hadjighassem M., Hosseindoost S. (2025). Ferroptosis as a therapeutic target in glioblastoma: Mechanisms and emerging strategies. Mol. Ther. Nucleic Acids.

[20] Sun H., Zhang J., Qi H., Jiang D., Hu C., Mao C., Liu W., Qi H., Zong J. (2025). Ioning out glioblastoma: Ferroptosis mechanisms and therapeutic frontiers. Cell Death Discov..

[21] Singh S., Mohapatra I., Barik D., Zheng H., Kim S., Sharma M., Chen C.C., Singh G. (2025). Harnessing ferroptosis to transform glioblastoma therapy and surmount treatment resistance. Cell Death Discov..

[22] Bo Y., Mu L., Yang Z., Li W., Jin M. (2024). Research progress on ferroptosis in gliomas (Review). Oncol. Lett..

[23] Zheng X., Diao M., Tong S., Yang S., Lin J., Zhuo S., Wang T., Dai J., Chen S., Wang K. (2025). Global research landscape and hotspots for ferroptosis in glioma: A comprehensive bibliometric and visual analysis. Heliyon.

[24] Sun Q., Lu H., Yuan F., Zhao Q., Wei Y., Wang R., Chen Q., Liu B. (2025). SLC10A3 regulates ferroptosis of glioblastoma through the STAT3/GPX4 pathway. Sci. Rep..

[25] Alborzinia H., Flórez A.F., Kreth S., Brückner L.M., Yildiz U., Gartlgruber M., Odoni D.I., Poschet G., Garbowicz K., Shao C. (2022). MYCN mediates cysteine addiction and sensitizes neuroblastoma to ferroptosis. Nat. Cancer.

[26] Alborzinia H., Chen Z., Yildiz U., Freitas F.P., Vogel F.C.E., Varga J.P., Batani J., Bartenhagen C., Schmitz W., Büchel G. (2023). LRP8-mediated selenocysteine uptake is a targetable vulnerability in MYCN-amplified neuroblastoma. EMBO Mol. Med..

[27] Lu Y., Yang Q., Su Y., Ji Y., Li G., Yang X., Xu L., Lu Z., Dong J., Wu Y. (2021). MYCN mediates TFRC-dependent ferroptosis and reveals vulnerabilities in neuroblastoma. Cell Death Dis..

[28] Xue X., Wang M., Cui J., Yang M., Ma L., Kang R., Tang D., Wang J. (2025). Glutathione metabolism in ferroptosis and cancer therapy. Cancer Lett..

[29] Chi H., Li B., Wang Q., Gao Z., Feng B., Xue H., Li G. (2023). Opportunities and challenges related to ferroptosis in glioma and neuroblastoma. Front. Oncol..

[30] Jiang X., Stockwell B.R., Conrad M. (2021). Ferroptosis: Mechanisms, biology and role in disease. Nat. Rev. Mol. Cell Biol..

[31] Liang D., Minikes A.M., Jiang X. (2022). Ferroptosis at the intersection of lipid metabolism and cellular signaling. Mol. Cell.

[32] Sun D., Wang L., Wu Y., Yu Y., Yao Y., Yang H., Hao C. (2025). Lipid metabolism in ferroptosis: Mechanistic insights and therapeutic potential. Front. Immunol..

[33] Lee J., Roh J.L. (2025). Lipid metabolism in ferroptosis: Unraveling key mechanisms and therapeutic potential in cancer. Biochim. Biophys. Acta Rev. Cancer.

[34] Pope L.E., Dixon S.J. (2023). Regulation of ferroptosis by lipid metabolism. Trends Cell Biol..

[35] Battaglia A.M., Chirillo R., Aversa I., Sacco A., Costanzo F., Biamonte F. (2020). Ferroptosis and Cancer: Mitochondria Meet the “Iron Maiden” Cell Death. Cells.

[36] Gao M., Monian P., Pan Q., Zhang W., Xiang J., Jiang X. (2016). Ferroptosis is an autophagic cell death process. Cell Res..

[37] Lin Z., Liu J., Kang R., Yang M., Tang D. (2021). Lipid Metabolism in Ferroptosis. Adv. Biol..

[38] Dixon S.J., Lemberg K.M., Lamprecht M.R., Skouta R., Zaitsev E.M., Gleason C.E., Patel D.N., Bauer A.J., Cantley A.M., Yang W.S. (2012). Ferroptosis: An iron-dependent form of nonapoptotic cell death. Cell.

[39] Li J., Cao F., Yin H.L., Huang Z.J., Lin Z.T., Mao N., Sun B., Wang G. (2020). Ferroptosis: Past, present and future. Cell Death Dis..

[40] Doll S., Freitas F.P., Shah R., Aldrovandi M., da Silva M.C., Ingold I., Goya Grocin A., Xavier da Silva T.N., Panzilius E., Scheel C.H. (2019). FSP1 is a glutathione-independent ferroptosis suppressor. Nature.

[41] Bersuker K., Hendricks J.M., Li Z., Magtanong L., Ford B., Tang P.H., Roberts M.A., Tong B., Maimone T.J., Zoncu R. (2019). The CoQ oxidoreductase FSP1 acts parallel to GPX4 to inhibit ferroptosis. Nature.

[42] Zhou Y., Zeng L., Cai L., Zheng W., Liu X., Xiao Y., Jin X., Bai Y., Lai M., Li H. (2025). Cellular senescence-associated gene IFI16 promotes HMOX1-dependent evasion of ferroptosis and radioresistance in glioblastoma. Nat. Commun..

[43] Qiu C., Zhang X., Huang B., Wang S., Zhou W., Li C., Li X., Wang J., Yang N. (2020). Disulfiram, a Ferroptosis Inducer, Triggers Lysosomal Membrane Permeabilization by Up-Regulating ROS in Glioblastoma. Onco Targets Ther..

[44] Chen T.C., Chuang J.Y., Ko C.Y., Kao T.J., Yang P.Y., Yu C.H., Liu M.S., Hu S.L., Tsai Y.T., Chan H. (2020). AR ubiquitination induced by the curcumin analog suppresses growth of temozolomide-resistant glioblastoma through disrupting GPX4-Mediated redox homeostasis. Redox Biol..

[45] Li X., Zhang W., Xing Z., Hu S., Zhang G., Wang T., Wang T., Fan Q., Chen G., Cheng J. (2024). Targeting SIRT3 sensitizes glioblastoma to ferroptosis by promoting mitophagy and inhibiting SLC7A11. Cell Death Dis..

[46] Cai J., Ye Z., Hu Y., Ye L., Gao L., Wang Y., Sun Q., Tong S., Zhang S., Wu L. (2023). Fatostatin induces ferroptosis through inhibition of the AKT/mTORC1/GPX4 signaling pathway in glioblastoma. Cell Death Dis..

[47] Wang Z., Wang Y., Shen N., Liu Y., Xu X., Zhu R., Jiang H., Wu X., Wei Y., Tang J. (2024). AMPKα1-mediated ZDHHC8 phosphorylation promotes the palmitoylation of SLC7A11 to facilitate ferroptosis resistance in glioblastoma. Cancer Lett..

[48] Meng X., Wang Z., Yang Q., Liu Y., Gao Y., Chen H., Li A., Li R., Wang J., Sun G. (2024). Intracellular C5aR1 inhibits ferroptosis in glioblastoma through METTL3-dependent m6A methylation of GPX4. Cell Death Dis..

[49] Zeng Y., Zhao L., Zeng K., Zhan Z., Zhan Z., Li S., Zhan H., Chai P., Xie C., Ding S. (2025). TRAF3 loss protects glioblastoma cells from lipid peroxidation and immune elimination via dysregulated lipid metabolism. J. Clin. Investig..

[50] Li X., Hu J., Zheng W., Fan Z., Chi H., Li H., Wang Y., Jing Z. (2025). CircMAN1A2 Levels Determine GBM Susceptibility to TMZ in a Pathway Involving TEP1- and KEAP1-Mediated NRF2 Degradation Leading to Ferroptosis. CNS Neurosci. Ther..

[51] Tian Q., Dan G., Wang X., Zhu J., Chen C., Tang D., Wang Z., Chen D., Lei S., Yang C. (2025). IDO1 inhibits ferroptosis by regulating FTO-mediated m6A methylation and SLC7A11 mRNA stability during glioblastoma progression. Cell Death Discov..

[52] Zhan S., Lu L., Pan S.S., Wei X.Q., Miao R.R., Liu X.H., Xue M., Lin X.K., Xu H.L. (2022). Targeting NQO1/GPX4-mediated ferroptosis by plumbagin suppresses in vitro and in vivo glioma growth. Br. J. Cancer.

[53] Zhou Y., Zhao Z., Jiang C., Nie C., Xiao D., Wu Z., Yu H., Zheng J., Wang X., Jiang X. (2025). LINC01088 prevents ferroptosis in glioblastoma by enhancing SLC7A11 via HLTF/USP7 axis. Clin. Transl. Med..

[54] Gao W., Li Y., Lin X., Deng K., Long X., Li D., Huang M., Wang X., Xu Y., She X. (2024). Procyanidin B1 Promotes PSMC3-NRF2 Ubiquitination to Induce Ferroptosis in Glioblastoma. Phytother. Res..

[55] Yang Y.H., Li W., Ren L.W., Yang H., Zhang Y.Z., Zhang S., Hao Y., Yu D.K., Tong R.S., Du G.H. (2024). S670, an amide derivative of 3-O-acetyl-11-keto-β-boswellic acid, induces ferroptosis in human glioblastoma cells by generating ROS and inhibiting STX17-mediated fusion of autophagosome and lysosome. Acta Pharmacol. Sin..

[56] Zhang K., Wu Y., Chen G., Wang H., Liu Y., Zhou Y. (2023). Heat shock protein 27 deficiency promotes ferrous ion absorption and enhances acyl-Coenzyme A synthetase long-chain family member 4 stability to promote glioblastoma cell ferroptosis. Cancer Cell Int..

[57] Huan R., Zhang J., Yue J., Yang S., Han G., Cheng Y., Tan Y. (2024). Orexin-A mediates glioblastoma proliferation inhibition by increasing ferroptosis triggered by unstable iron pools and GPX4 depletion. J. Cell. Mol. Med..

[58] Liu Y., Jiang N., Chen W., Zhang W., Shen X., Jia B., Chen G. (2024). TRIM59-mediated ferroptosis enhances neuroblastoma development and chemosensitivity through p53 ubiquitination and degradation. Heliyon.

[59] Li Q., Cheng Y., Yang C., Tian M., Wang X., Li D., Li X., Qu J., Zhou S., Zheng L. (2025). Targeting the Exonic Circular OGT RNA/O-GlcNAc Transferase/Forkhead Box C1 Axis Inhibits Asparagine- and Alanine-Mediated Ferroptosis Repression in Neuroblastoma Progression. Research.

[60] Valenti G.E., Roveri A., Venerando R., Menichini P., Monti P., Tasso B., Traverso N., Domenicotti C., Marengo B. (2023). PTC596-Induced BMI-1 Inhibition Fights Neuroblastoma Multidrug Resistance by Inducing Ferroptosis. Antioxidants.

[61] Li Z., Wang Y., Liang S., Yuan T., Liu J. (2024). EIF2S1 Silencing Impedes Neuroblastoma Development Through GPX4 Inactivation and Ferroptosis Induction. Int. J. Genom..

[62] Chen Z., Inague A., Kaushal K., Fazeli G., Schilling D., Xavier da Silva T.N., Dos Santos A.F., Cheytan T., Freitas F.P., Yildiz U. (2024). PRDX6 contributes to selenocysteine metabolism and ferroptosis resistance. Mol. Cell.

[63] Qiu L., Zhou R., Luo Z., Wu J., Jiang H. (2022). CDC27-ODC1 Axis Promotes Metastasis, Accelerates Ferroptosis and Predicts Poor Prognosis in Neuroblastoma. Front. Oncol..

[64] Freitas F.P., Alborzinia H., Dos Santos A.F., Nepachalovich P., Pedrera L., Zilka O., Inague A., Klein C., Aroua N., Kaushal K. (2024). 7-Dehydrocholesterol is an endogenous suppressor of ferroptosis. Nature.

[65] Hassannia B., Wiernicki B., Ingold I., Qu F., Van Herck S., Tyurina Y.Y., Bayır H., Abhari B.A., Angeli J.P.F., Choi S.M. (2018). Nano-targeted induction of dual ferroptotic mechanisms eradicates high-risk neuroblastoma. J. Clin. Investig..

[66] Seneviratne J.A., Carter D.R., Mittra R., Gifford A., Kim P.Y., Luo J.S., Mayoh C., Salib A., Rahmanto A.S., Murray J. (2023). Inhibition of mitochondrial translocase SLC25A5 and histone deacetylation is an effective combination therapy in neuroblastoma. Int. J. Cancer.

[67] Sun Q., Xu Y., Yuan F., Qi Y., Wang Y., Chen Q., Liu B. (2022). Rho family GTPase 1 (RND1), a novel regulator of p53, enhances ferroptosis in glioblastoma. Cell Biosci..

[68] Zheng X., Zhang C. (2023). The Regulation of Ferroptosis by Noncoding RNAs. Int. J. Mol. Sci..

[69] Shenoy G., Connor J.R. (2023). A closer look at the role of iron in glioblastoma. Neuro Oncol..

[70] Lathoria K., Gowda P., Umdor S.B., Patrick S., Suri V., Sen E. (2023). PRMT1 driven PTX3 regulates ferritinophagy in glioma. Autophagy.

[71] Mohan M., Mannan A., Kakkar C., Singh T.G. (2025). Nrf2 and Ferroptosis: Exploring Translational Avenues for Therapeutic Approaches to Neurological Diseases. Curr. Drug Targets.

[72] Lv G., Li X., Deng H., Zhang J., Gao X. (2024). Regulatory Mechanisms of STAT3 in GBM and its Impact on TMZ Resistance. Curr. Mol. Pharmacol..

[73] Xue L., Luo K., Hou K., Huo W., Ruan P., Xue Y., Yao X., Meng C., Xia D., Tang Y. (2024). Targeted Gold Nanoclusters for Synergistic High-Risk Neuroblastoma Therapy through Noncanonical Ferroptosis. ACS Appl. Mater. Interfaces.

[74] Agostini M., Melino G., Habeb B., Calandria J.M., Bazan N.G. (2022). Targeting lipid metabolism in cancer: Neuroblastoma. Cancer Metastasis Rev..

[75] Cioffi G., Waite K.A., Edelson J.L., Kruchko C., Ostrom Q.T., Barnholtz-Sloan J.S. (2022). Changes in survival over time for primary brain and other CNS tumors in the United States, 2004-2017. J. Neurooncol..

[76] Zhang Q., Yu H., Zhong J., Cheng W., Qi Y. (2025). Global, regional, and national burden of brain and central nervous system cancer: A systematic analysis of incidence, deaths, and DALYS with predictions to 2040. Int. J. Surg..

[77] Deng S., Zheng Y., Mo Y., Xu X., Li Y., Zhang Y., Liu J., Chen J., Tian Y., Ke Y. (2021). Ferroptosis Suppressive Genes Correlate with Immunosuppression in Glioblastoma. World Neurosurg..

[78] Lv Y., Gao Y., Di W., Li Z., Shi Y., Hou T., Chen Y., Tian J., Xu M., Su W. (2025). MFAP4 is a novel prognostic biomarker in glioma correlating with immunotherapy resistance and ferroptosis. Front. Pharmacol..

[79] Wu Y., Liu L., Li Z., Zhang T., Wang Q., Cheng M. (2025). A Risk Model Based on Ferroptosis-Related Genes OSMR, G0S2, IGFBP6, IGHG2, and FMOD Predicts Prognosis in Glioblastoma Multiforme. CNS Neurosci. Ther..

[80] Fu H., Zhang Z., Li D., Lv Q., Chen S., Zhang Z., Wu M. (2022). LncRNA PELATON, a Ferroptosis Suppressor and Prognositic Signature for GBM. Front. Oncol..

[81] Wan R.J., Peng W., Xia Q.X., Zhou H.H., Mao X.Y. (2021). Ferroptosis-related gene signature predicts prognosis and immunotherapy in glioma. CNS Neurosci. Ther..

[82] Cheng H., Ling F., Hou X., Wang J., Zhao Y., Wang Y., Cao Y. (2024). The Role of STEAP3 in Pathogenesis of Gliomas: An Independent Prognostic Factor and Regulator of Ferroptosis. Ann. Clin. Lab. Sci..

[83] Chen Y., Li Z., Cao Q., Guan H., Mao L., Zhao M. (2022). Ferroptosis-related gene signatures in neuroblastoma associated with prognosis. Front. Cell Dev. Biol..

[84] Tan L., He G., Shen C., He S., Chen Y., Guo X. (2025). Construction of a ferroptosis-based prediction model for the prognosis of MYCN-amplified neuroblastoma and screening and verification of target sites. Hereditas.

[85] Chu J. (2025). Study of an N6-methyladenosine- and ferroptosis-related prognostic model and the mechanisms underlying the molecular network in neuroblastoma based on multiple datasets. Discov. Oncol..

[86] Cheng J., Dong X., Yang Y., Qin X., Zhou X., Zhang D. (2024). Synergistic machine learning models utilizing ferroptosis-related genes for improved neuroblastoma outcome prediction. Transl. Pediatr..

[87] Sareen H., Ma Y., Becker T.M., Roberts T.L., de Souza P., Powter B. (2022). Molecular Biomarkers in Glioblastoma: A Systematic Review and Meta-Analysis. Int. J. Mol. Sci..

[88] Liu T., Zhu C., Chen X., Guan G., Zou C., Shen S., Wu J., Wang Y., Lin Z., Chen L. (2022). Ferroptosis, as the most enriched programmed cell death process in glioma, induces immunosuppression and immunotherapy resistance. Neuro Oncol..

[89] Feng W., Liu Y., Zhang Q., Hu S., Xie D., Tan P., Lei Y., Chen C., Ren C., Du S. (2025). GDF15 Drives Glioblastoma Radioresistance by Inhibiting Ferroptosis and Remodeling the Immune Microenvironment. Int. J. Biol. Sci..

[90] Galli S., Naranjo A., Van Ryn C., Tilan J.U., Trinh E., Yang C., Tsuei J., Hong S.H., Wang H., Izycka-Swieszewska E. (2016). Neuropeptide Y as a Biomarker and Therapeutic Target for Neuroblastoma. Am. J. Pathol..

[91] Cohn S.L., Pearson A.D., London W.B., Monclair T., Ambros P.F., Brodeur G.M., Faldum A., Hero B., Iehara T., Machin D. (2009). The International Neuroblastoma Risk Group (INRG) classification system: An INRG Task Force report. J. Clin. Oncol..

[92] Tan S., Hou X., Mei L. (2020). Dihydrotanshinone I inhibits human glioma cell proliferation via the activation of ferroptosis. Oncol. Lett..

[93] Lu S., Wang X.Z., He C., Wang L., Liang S.P., Wang C.C., Li C., Luo T.F., Feng C.S., Wang Z.C. (2021). ATF3 contributes to brucine-triggered glioma cell ferroptosis via promotion of hydrogen peroxide and iron. Acta Pharmacol. Sin..

[94] Lv D., Zhong C., Dixit D., Yang K., Wu Q., Godugu B., Prager B.C., Zhao G., Wang X., Xie Q. (2023). EGFR promotes ALKBH5 nuclear retention to attenuate N6-methyladenosine and protect against ferroptosis in glioblastoma. Mol. Cell.

[95] Kim N.Y., Shivanne Gowda S.G., Lee S.G., Sethi G., Ahn K.S. (2024). Cannabidiol induces ERK activation and ROS production to promote autophagy and ferroptosis in glioblastoma cells. Chem. Biol. Interact..

[96] Zheng X.J., Chen W.L., Yi J., Li W., Liu J.Y., Fu W.Q., Ren L.W., Li S., Ge B.B., Yang Y.H. (2022). Apolipoprotein C1 promotes glioblastoma tumorigenesis by reducing KEAP1/NRF2 and CBS-regulated ferroptosis. Acta Pharmacol. Sin..

[97] Chen Y., Mi Y., Zhang X., Ma Q., Song Y., Zhang L., Wang D., Xing J., Hou B., Li H. (2019). Dihydroartemisinin-induced unfolded protein response feedback attenuates ferroptosis via PERK/ATF4/HSPA5 pathway in glioma cells. J. Exp. Clin. Cancer Res..

[98] Zhang Y., Kong Y., Ma Y., Ni S., Wikerholmen T., Xi K., Zhao F., Zhao Z., Wang J., Huang B. (2021). Loss of COPZ1 induces NCOA4 mediated autophagy and ferroptosis in glioblastoma cell lines. Oncogene.

[99] Wang W., Zhang Y., Li X., E Q., Jiang Z., Shi Q., Huang Y., Wang J., Huang Y. (2024). KCNA1 promotes the growth and invasion of glioblastoma cells through ferroptosis inhibition via upregulating SLC7A11. Cancer Cell Int..

[100] Li C., Jing J., Wang Y., Jiang H. (2024). CENPA facilitates glioma stem cell stemness and suppress ferroptosis to accelerate glioblastoma multiforme progression by promoting GBP2 transcription. Pathol. Res. Pract..

[101] Wang Y., Gao Q., Chen X., Dong Q., Luan R., Li F., Lu H., Zhou X. (2025). TCF4 Promotes Neuroblastoma Proliferation and Inhibits Ferroptosis by Transactivating GPX4 Expression. Appl. Biochem. Biotechnol..

[102] Faraji P., Borchert A., Ahmadian S., Kuhn H. (2024). Butylated Hydroxytoluene (BHT) Protects SH-SY5Y Neuroblastoma Cells from Ferroptotic Cell Death: Insights from In Vitro and In Vivo Studies. Antioxidants.

[103] Shir J.C., Chen P.Y., Kuo C.H., Hsieh C.H., Chang H.Y., Lee H.C., Huang C.H., Hsu C.H., Hsu W.M., Huang H.C. (2025). DHODH Blockade Induces Ferroptosis in Neuroblastoma by Modulating the Mevalonate Pathway. Mol. Cell. Proteom..

[104] Lu Q., Lu X., Zhang Y., Huang W., Zhou H., Li T. (2022). Recent advances in ferroptosis and therapeutic strategies for glioblastoma. Front. Mol. Biosci..

[105] Zhuo S., He G., Chen T., Li X., Liang Y., Wu W., Weng L., Feng J., Gao Z., Yang K. (2022). Emerging role of ferroptosis in glioblastoma: Therapeutic opportunities and challenges. Front. Mol. Biosci..

[106] Yang E., Wang L., Jin W., Liu X., Wang Q., Wu Y., Tan Y., Wang Y., Cui X., Zhao J. (2022). PTRF/Cavin-1 enhances chemo-resistance and promotes temozolomide efflux through extracellular vesicles in glioblastoma. Theranostics.

[107] Qu S., Qi S., Zhang H., Li Z., Wang K., Zhu T., Ye R., Zhang W., Huang G., Yi G.Z. (2023). Albumin-bound paclitaxel augment temozolomide treatment sensitivity of glioblastoma cells by disrupting DNA damage repair and promoting ferroptosis. J. Exp. Clin. Cancer Res..

[108] Li J., Wu Y., Wang J., Xu X., Zhang A., Li Y., Zhang Z. (2023). Macrophage Membrane-Coated Nano-Gemcitabine Promotes Lymphocyte Infiltration and Synergizes AntiPD-L1 to Restore the Tumoricidal Function. ACS Nano.

[109] Cao Z., Liu X., Zhang W., Zhang K., Pan L., Zhu M., Qin H., Zou C., Wang W., Zhang C. (2023). Biomimetic Macrophage Membrane-Camouflaged Nanoparticles Induce Ferroptosis by Promoting Mitochondrial Damage in Glioblastoma. ACS Nano.

[110] Liu Y., Fleishman J.S., Wang H., Huo L. (2025). Pharmacologically Targeting Ferroptosis and Cuproptosis in Neuroblastoma. Mol. Neurobiol..

[111] Pitts M.G., Bryant L.T., Buoncristiani M.D., Rellinger E.J. (2025). MYCN-Driven Metabolic Networks Are a Critical Dependency of High-Risk Neuroblastomas. Cancers.

[112] Dahlmanns M., Yakubov E., Dahlmanns J.K. (2021). Genetic Profiles of Ferroptosis in Malignant Brain Tumors and Off-Target Effects of Ferroptosis Induction. Front. Oncol..

[113] Gnanamony M., Thomas M., Nguyen T.H., Brownstein K., de Alarcon P.A. (2025). Pomiferin Induces Antiproliferative and Pro-Death Effects in High-Risk Neuroblastoma Cells by Modulating Multiple Cell Death Pathways. Int. J. Mol. Sci..

[114] Zhang X., Fryknäs M., Hernlund E., Fayad W., De Milito A., Olofsson M.H., Gogvadze V., Dang L., Påhlman S., Schughart L.A. (2014). Induction of mitochondrial dysfunction as a strategy for targeting tumour cells in metabolically compromised microenvironments. Nat. Commun..

[115] Liu R., Shi P., Wang Z., Yuan C., Cui H. (2020). Molecular Mechanisms of MYCN Dysregulation in Cancers. Front. Oncol..

[116] Jakobsson A.W., Kundu S., Guo J., Chowdhury A., Zhao M., Lindell E., Bergsten P., Swartling F.J., Sjöblom T., Zhang X. (2022). Iron Chelator VLX600 Inhibits Mitochondrial Respiration and Promotes Sensitization of Neuroblastoma Cells in Nutrition-Restricted Conditions. Cancers.

[117] Geng N., Shi B.J., Li S.L., Zhong Z.Y., Li Y.C., Xua W.L., Zhou H., Cai J.H. (2018). Knockdown of ferroportin accelerates erastin-induced ferroptosis in neuroblastoma cells. Eur. Rev. Med. Pharmacol. Sci..

[118] Wang Y.Q., Chang S.Y., Wu Q., Gou Y.J., Jia L., Cui Y.M., Yu P., Shi Z.H., Wu W.S., Gao G. (2016). The Protective Role of Mitochondrial Ferritin on Erastin-Induced Ferroptosis. Front. Aging Neurosci..

[119] Zhou Q., Meng Y., Li D., Yao L., Le J., Liu Y., Sun Y., Zeng F., Chen X., Deng G. (2024). Ferroptosis in cancer: From molecular mechanisms to therapeutic strategies. Signal Transduct. Target. Ther..

[120] Nie Z., Chen M., Gao Y., Huang D., Cao H., Peng Y., Guo N., Wang F., Zhang S. (2022). Ferroptosis and Tumor Drug Resistance: Current Status and Major Challenges. Front. Pharmacol..

[121] Greaves M., Maley C.C. (2012). Clonal evolution in cancer. Nature.

[122] Zhang C., Liu X., Jin S., Chen Y., Guo R. (2022). Ferroptosis in cancer therapy: A novel approach to reversing drug resistance. Mol. Cancer.

[123] Niu X., You Q., Hou K., Tian Y., Wei P., Zhu Y., Gao B., Ashrafizadeh M., Aref A.R., Kalbasi A. (2025). Autophagy in cancer development, immune evasion, and drug resistance. Drug Resist. Updat..

[124] Alatawi A.D., Venkatesan K., Asseri K., Paulsamy P., Alqifari S.F., Ahmed R., Nagoor Thangam M.M., Sirag N., Qureshi A.A., Elsayes H.A. (2025). Targeting Ferroptosis in Rare Neurological Disorders Including Pediatric Conditions: Innovations and Therapeutic Challenges. Biomedicines.

[125] Zhang L., Luo Y.L., Xiang Y., Bai X.Y., Qiang R.R., Zhang X., Yang Y.L., Liu X.L. (2024). Ferroptosis inhibitors: Past, present and future. Front. Pharmacol..

[126] Stepanić V., Kučerová-Chlupáčová M. (2023). Review and Chemoinformatic Analysis of Ferroptosis Modulators with a Focus on Natural Plant Products. Molecules.

[127] Yan D., Wu Z., Qi X. (2023). Ferroptosis-related metabolic mechanism and nanoparticulate anticancer drug delivery systems based on ferroptosis. Saudi Pharm. J..

[128] Wang Y., Sun T., Jiang C. (2022). Nanodrug delivery systems for ferroptosis-based cancer therapy. J. Control. Release.

[129] Amreddy N., Babu A., Muralidharan R., Panneerselvam J., Srivastava A., Ahmed R., Mehta M., Munshi A., Ramesh R. (2018). Recent Advances in Nanoparticle-Based Cancer Drug and Gene Delivery. Adv. Cancer Res..

[130] Chen S., Shi J., Yu D., Dong S. (2024). Advance on combination therapy strategies based on biomedical nanotechnology induced ferroptosis for cancer therapeutics. Biomed. Pharmacother..

[131] Chen H., Lyu F., Gao X. (2024). Advances in ferroptosis for castration-resistant prostate cancer treatment: Novel drug targets and combination therapy strategies. Prostate Cancer Prostatic Dis..

[132] Cui K., Wang K., Huang Z. (2024). Ferroptosis and the tumor microenvironment. J. Exp. Clin. Cancer Res..

[133] Wolf A., Leardini D., Li L., Masetti R., Lyssiotis C.A., Barbieri E. (2025). Deciphering the MYCN-driven metabolic microenvironment of neuroblastoma. Trends Mol. Med..

[134] Duan M., Cao R., Yang Y., Chen X., Liu L., Ren B., Wang L., Goh B.C. (2024). Blood-Brain Barrier Conquest in Glioblastoma Nanomedicine: Strategies, Clinical Advances, and Emerging Challenges. Cancers.

[135] Khan A., Huo Y., Guo Y., Shi J., Hou Y. (2024). Ferroptosis is an effective strategy for cancer therapy. Med. Oncol..

[136] Wei Y., Duan S., Gong F., Li Q. (2022). The RNA-binding protein fragile-X mental retardation autosomal 1 (FXR1) modulates glioma cells sensitivity to temozolomide by regulating ferroptosis. Biochem. Biophys. Res. Commun..

[137] Ye L.F., Chaudhary K.R., Zandkarimi F., Harken A.D., Kinslow C.J., Upadhyayula P.S., Dovas A., Higgins D.M., Tan H., Zhang Y. (2020). Radiation-Induced Lipid Peroxidation Triggers Ferroptosis and Synergizes with Ferroptosis Inducers. ACS Chem. Biol..

[138] Fernández-Acosta R., Vintea I., Koeken I., Hassannia B., Vanden Berghe T. (2025). Harnessing ferroptosis for precision oncology: Challenges and prospects. BMC Biol..

[139] Yang H., Yao X., Liu Y., Shen X., Li M., Luo Z. (2023). Ferroptosis Nanomedicine: Clinical Challenges and Opportunities for Modulating Tumor Metabolic and Immunological Landscape. ACS Nano.

